# Mesh Or Patch for Hernia on Epigastric and Umbilical Sites (MORPHEUS trial): study protocol for a multi-centre patient blinded randomized controlled trial

**DOI:** 10.1186/1471-2482-14-33

**Published:** 2014-05-22

**Authors:** Jeroen EH Ponten, Bart JM Leenders, Jan A Charbon, Tanja Lettinga - van de Poll, Jeroen Heemskerk, Ingrid S Martijnse, Joop LM Konsten, Simon W Nienhuijs

**Affiliations:** 1 Departement of Surgery, Catharina Ziekenhuis Eindhoven, Michelangelolaan 2, 5623 EJ Eindhoven, The Netherlands; 2 Departement of Surgery, Maxima Medisch Centrum, De Run 4600, 5504 DB Veldhoven, The Netherlands; 3 Departement of Surgery, St. Jans Gasthuis Weert, Vogelsbleek 5, 6001 BE Weert, The Netherlands; 4 Departement of Surgery, Laurentius Ziekenhuis Roermond, Monseigneur Driessenstraat 6, 6043 CV Roermond, The Netherlands; 5 Departement of Surgery, TweeSteden Ziekenhuis Tilburg, Dr. Deelenlaan 5, 5042 AD Tilburg, The Netherlands; 6 Departement of Surgery, VieCuri Medisch Centrum, Tegelseweg 210, 5912 BL Venlo, The Netherlands

**Keywords:** Umbilical, Epigastric, Hernia, Herniorraphy, Mesh repair, Proceed Ventral Patch, Complications, Pain, Recurrence, Costs

## Abstract

**Background:**

Evidence is accumulating that, similar to other ventral hernias, umbilical and epigastric hernias must be mesh repaired. The difficulties involved in mesh placement and in mesh-related complications could be the reason many small abdominal hernias are still primary closed. In laparoscopic repair, a mesh is placed intraperitoneally, while the most common procedure is open surgery is pre-peritoneal mesh placement. A recently developed alternative method is the so-called patch repair, in this approach a mesh can be placed intraperitoneally through open surgery. In theory, such patches are particularly suitable for small hernias due to a reduction in the required dissection. This simple procedure is described in several studies. It is still unclear whether this new approach is associated with an equal risk of recurrence and complications compared with pre-peritoneal meshes. The material of the patch is in direct contact with intra-abdominal organs, it is unknown if this leads to more complications. On the other hand, the smaller dissection in the pre-peritoneal plane may lead to a reduction in wound complications.

**Methods/Design:**

346 patients suffering from an umbilical or epigastric hernia will be included in a multi-centre patient-blinded trial, comparing mesh repair with patch repair. Randomisation will take place for the two operation techniques. The two devices investigated are a flat pre-peritoneal mesh and a Proceed Ventral Patch®. Stratification will occur per centre. Post-operative evaluation will take place after 1, 3, 12 and 24 months. The number of complications requiring treatment is the primary endpoint. Secondary endpoints are Verbal Descriptor Scale (VDS) pain score and VDS cosmetic score, operation duration, recurrence and costs. An intention to treat analysis will be performed.

**Discussion:**

This trial is one of the first in its kind, to compare different mesh devices in a randomized controlled setting. The results will help to evaluate mesh repair for epigastric an umbilical hernia, and find a surgical method that minimizes the complication rate.

**Trial registration:**

Netherlands Trail Registration (NTR) www.trialregister.nl 2010 NTR2514 NL33995.060.10

## Background

The evidence that umbilical and epigastric hernias must be repaired using a mesh, in analogy with other hernias, is accumulating [1-7]. The use of a mesh in large hernias reduces the risk of recurrence from approximately 15-40% to 1-10% [1,4-6,8-10]. However, less research has been performed if this also applies for small hernias. To date, most small hernias are still closed with primary sutures, because of the sometimes-difficult pre-peritoneal plane in which the mesh should be placed. Therefore, the results of the Hernia Umbilicalis Mesh versus Primair suture (HUMP) trial that focuses on hernias smaller than 2 centimetres will have great added value for umbilical hernias in particular [11]. Other retrospective studies suggest that for the reoperation rate due to recurrence mesh is superior in umbilical and epigastric hernias [7]. In contrast to the HUMP trial, which places the mesh in this pre-peritoneal plane, a mesh can also be placed intraperitoneally. Advantages of intraperitoneal mesh placement can be shorter operation time. Intraperitoneal mesh placement can also be done by a laparoscopic approach [12-14]. Although in the laparoscopic mesh placement, several new fascia openings in the abdominal wall are created. The search for alternative techniques without these additional fascia defects may have played an important role in the development of these so-called patches. Patches are small devices, which can be inserted by open surgery and placed underneath the peritoneum [15-17].

In theory, such patches are particularly suitable for small hernias because of the lesser dissection necessary. It is still unclear whether this simpler procedure is also associated with at least an equal risk of recurrence and complications compared to conventional mesh placement. The ease of a surgical procedure is not a clear clinically relevant parameter, but it does often play a significant role in the surgeon’s choices. Not only are there implications for approximately 4100 umbilical and 2400 epigastric operations in the Netherlands per year [18], but also for a frequently occurring other abdominal wall hernia, the small incisional hernia.

The MORPHEUS trial is designed to clarify which treatment for primary umbilical and epigastric hernias is superior in terms of complications, costs and recurrence.

## Methods

### Design

The MORPHEUS trial is a multicentre, randomized controlled, nationwide, patient-blinded, superiority study. Patients will be randomly allocated to undergo open flat mesh repair or open patch repair for primary umbilical or epigastric hernias. The aim of this study is to determine the ideal treatment for patients with these conditions in terms of operation related complication and postoperative pain.

### Objectives/Endpoints

The primary objective of the MORPHEUS trial is to evaluate whether a ventral patch is associated with fewer complications than in conventional mesh placement for epigastric and umbilical repair. A complication is defined as an undesired development that requires treatment. Complications will be recorded up to three months after the initial procedure and pain up to 24 months post-operative. Secondary endpoints are differences in recurrence, cosmetic score, operation duration and costs.

### Participants

The institutional review board of each participating hospital has approved this randomized controlled trial. Inclusion started February the 1^st^ 2011. Adult patients with a single primary umbilical or epigastric hernia qualify for participation in the study. Patients with incarcerated hernia are also included. The maximum size of the herniation orifice can be 3 cm, corresponding with 2 fingers in analogy with the European Herniation Society (EHS) classification for inguinal hernias [19]. Patients with diagnosed ascites are excluded. This also applies for patients who cannot sufficiently understand and/or follow through participation in a trial. For the related selection criteria see Table 1.

**Table 1:** Inclusion criteria

**Hernia related**	**Patient related**
In epigastric or (peri-) umbilical region	Age 18 years or older
< 3 cm (2 fingers)	Capacity (comprehension, language ability and physical ability)
Primary	No ascites
Single hernia	

Possible participants receive verbal and written information about the trial, which includes an informed consent form. Those who participate must sign the informed consent form, which must also be signed by the investigator. If a patient does not participate in the trial, the reason for this must be stated. A patient included in the trial is requested to fill in a questionnaire (P1) before the operation.

### Interventions

All procedures will take place under general anaesthesia. Administering a local anaesthetic peri-operatively is recommended. Prophylactic antibiotics are given on indication only. The use of a steri-drape as well as drains is not advised. Enlarging the herniation orifice as well as closing fascia over the mesh is permitted for both techniques.

The conventional mesh procedure begins with a para-umbilical or median incision across the hernia, then dissection of the fascia and mobilisation of the hernia sac. Opening of the hernia sac for inspection is permitted. Dissection of the pre-peritoneal area takes place after repositioning of the hernia. A flat large pore and lightweight polypropylene mesh with a minimum diameter of 6 cm is placed. Fixation of the mesh is carried out with non-absorbable monofilament sutures.

For the patch procedure, the hernia sac is opened. Possible adhesions must be released intraperitoneally. The patch is placed underneath the peritoneum, the slips fixed to the fascia. If in the surgeon’s opinion the hernia sac can be repositioned without opening it, placement is also permitted in the pre-peritoneal plane. We have chosen the standard of a PROCEED™ VENTRAL PATCH (Ethicon, Norderstedt, Germany) for this trial. No specific brand was chosen for the flat large pore and lightweight polypropylene mesh.

### Follow-up and flow chart protocol

**Figure 1:**
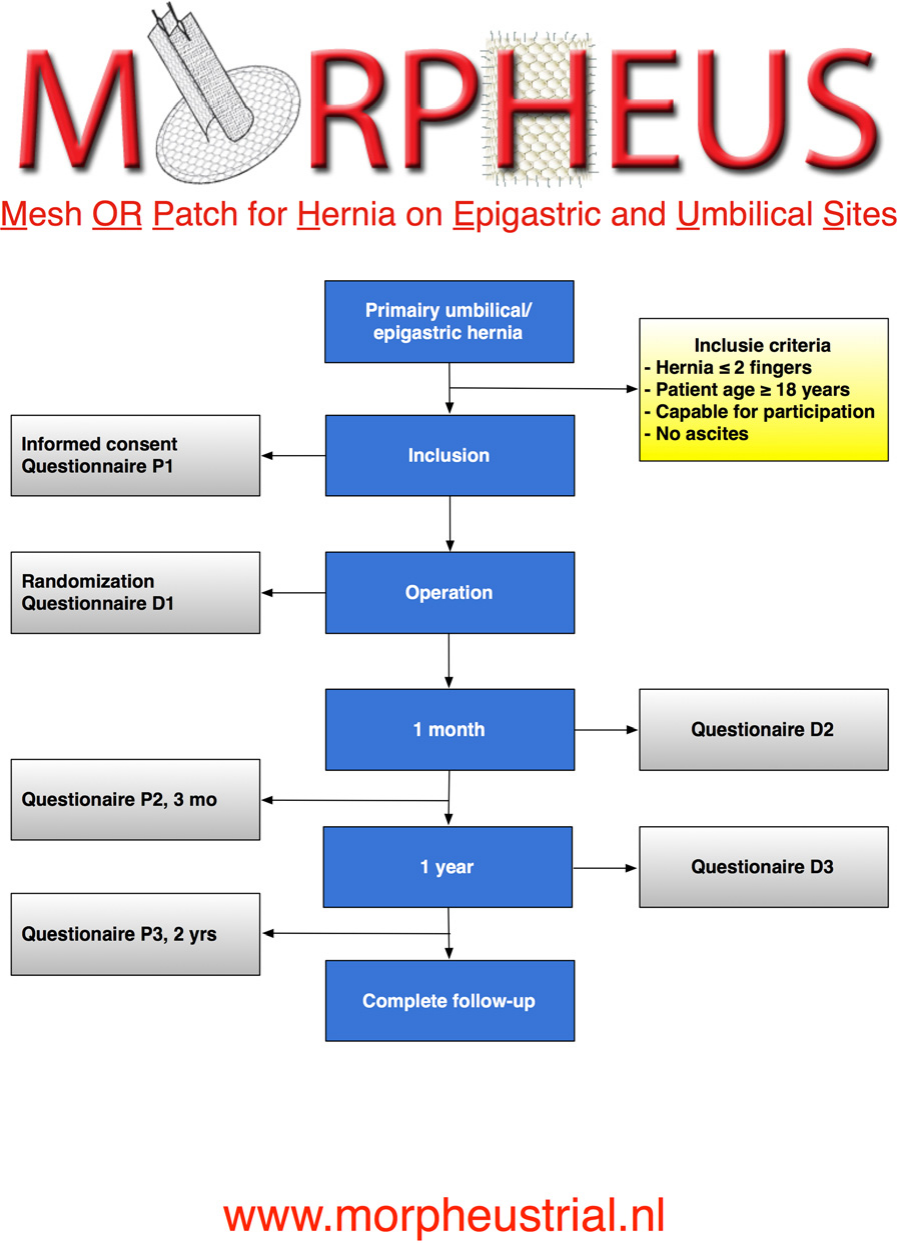
Trial flow-chart.

The surgeon must fill in a questionnaire (D1) immediately after the operation. The patients are advised to take 1 gram of paracetamol 3 times a day for the first 2 weeks, and to begin mobilizing immediately according to their capacity. Follow-up at the outpatient clinic takes place after 1 and 12 months, with the practitioner filling in the questionnaires (D2 and D3). The patients are asked to fill in a questionnaire 3 and 24 months after the operation (P2 and P3). These questionnaires will be sent to the patients by postal service or e-mail. Non-responders were contacted up to three times by means of a telephone questionnaire. Figure 1 shows the studies flow-chart.

### Calculation of random sample size

The primary outcome is the number of complications that require treatment. These are defined in detail further in the protocol. The occurrence of complications up to 23% is described [4]. Trials involving patch repair report a much lower complication rate, but the number of trials is significantly less. It is assumed that a conventional procedure leads to a complication in 20% of the cases [4,20]. The hypothesis is that this is reduced to 10% if a patch is used for repair. At a significance level of 5% and a power of 90%, the random sample size is 137 patients per group in a bilateral t-test. In the result analysis a Mann–Whitney parametric test will be used to evaluate a normal distribution. Therefore, the sample size must be increased by 15% [21]. Taking a minimum of 10% loss-to-follow-up into account as well, the total number of patients to be randomized becomes 346.

### Participant recruitment and retention

The surgeon will recruit the patients. Participation is not stimulated in any way other than voluntary participation.

### Randomisation

Sequence generation and randomisation will be performed by http://www.random.org/. The generated sequence is printed on paper cards with randomization numbers. These cards are put in non-see through envelopes. These envelopes can only be opened in the operating theatre just before timeout procedure, without informing the patient about the outcome of randomisation

### Blinding

The trial is a patient-blinded type. The patient is informed on the type of mesh device used by means of the questionnaire after 24 months (P3). In the data handling phase of the, the annalist is blinded for treatment. The consulted physician during follow-up is not blind for the received treatment.

### Data outcome

A Verbal Descriptor Scale (VDS) is a scale of consecutive descriptions. The following terms are used in the VDS pain score, from which patients can choose to express the level of pain: None, Mild, Moderate, Severe.

#### Questionnaire P1 – by the patient – preoperative

Collected data: height, weight, VDS pain score at rest, pain score while exercising and VDS cosmetic score, comorbidity and daily workload.

#### Questionnaire D1 – by the doctor – peroperative

Collected data: randomised device used (mesh/patch), presence of incarceration, resection of the hernia protrusion, number of fingers for the width of the herniation orifice, the need of widening the herniation orifice, closure of fascia over mesh, presence of adhesions when placing the device intraperitoneally, operation duration, VDS ease, possible reasons protocol violation.

#### Questionnaire D2 – by the doctor – 1 month postoperative

Data collected are: complications, use of analgesics at that time, VDS pain score at rest, pain score while exercising and VDS cosmetic score.

#### Questionnaire P2 – by the patient – 3 months postoperative

Collected data: complications, recurrence, use of analgesics at that time, VDS pain score at rest, pain score while exercising and VDS cosmetic score.

#### Questionnaire D3 – by the doctor – 1 year postoperative

Collected data: recurrence, use of analgesics at that time, VDS pain score at rest, pain score while exercising and VDS cosmetic score.

#### Questionnaire P3 – by the patient – 2 years postoperative

Collected data: recurrence, use of analgesics at that time, VDS pain score at rest, pain score while exercising and VDS cosmetic score.

### Definitions

A complication is defined as an undesired development that requires treatment, related to the operation site, occurring within 3 months postoperative. Treatment could also been initiated by the general practitioner, or other than that by the hospital surgeon. Complications are defined as following:

Extending operation time due to bleeding or other injury during the procedure

Prescribed medication treatment such as antibiotics and pain killers other than paracetamol after discharge

Re-operation such as evacuation of a haematoma, drainage of an abscess, exploration related to pain or intra-abdominal problems, or early recurrence

Wound care which requires more than once a day irrigation of the wound

Hospitalisation longer than scheduled or re-admission

The costs will be calculated post-hoc on the basis of the number of visits to the outpatient clinic, operation duration, costs of prosthesis and complication-related costs up to 2 years after the operation.

### Data collection methods

Completed informed consent forms remain at a central location of the participating hospital. When participating and signing, the patient receives a copy and the original stays at the hospital.

Questionnaires P1 and P2 with return envelope are given to the patient when he or she visits the outpatient clinic. P3 (2 years postoperative) is sent from the coordinating research hospital.

Questionnaires for surgeons D1-3 are preferably entered in an Excel/Access database on a hard disk accessible in the hospital. This can also be a paper version if so desired.

### Data management

Completed informed consent forms must be faxed/scanned/e-mailed to the coordinating research hospital. The questionnaires use codes consisting of the first initial and the first 2 letters of the last name followed by the serial number of the participating hospital; this to secure anonymity.

Informed consent forms and questionnaires remain in one place at the participating hospital. A password is used for the input program for surgeons.

### Statistical method

A p-value <0.05 is regarded as significant. SPSS statistics will be used for processing.

Non-adherent data are not anticipated for this trial. Prevention of missing values will be handled by telephone checks. If any missing values remain, an analysis will be made of the cause (completely at random/at random/not at random). An intention to treat analysis will be performed.

Comparison between the two interventions can be subdivided in three periods; Pre-operative, per-operative en post-operative comparison.

### Pre-operative

Body Mass Index (BMI), VDS pain score at rest, pain score while exercising and VDS cosmetic score, comorbidity and daily workload. Differences in baseline characteristics will be measured and presented in a baseline characteristics table. The Mann–Whitney test is used for continuous data and the Pearson’s chi-square/Fisher’s exact test for categorical data.

### Per-operative

Presence of incarceration, resection of the hernia protrusion, number of fingers for the width of the herniation orifice, the need of widening the herniation orifice, closure of fascia over mesh, presence of adhesions when placing the device intraperitoneally, operation duration, VDS ease, possible reasons protocol violation. The Mann–Whitney test is used for continuous data and the Pearson’s chi-square/Fisher’s exact test for categorical data.

### Post-operative

Occurrence of complications and pain score at rest and pain score while exercising (VDS), the use of analgesics at that time, cosmetic (VDS) and signs of recurrence (1,3,12,24 months post-operative). The Mann–Whitney test is used for continuous data and the Pearson’s chi-square/Fisher’s exact test for categorical data. If possible a Spearman’s correlations are used for analysis of relations between the (sub) groups (comorbidity, chronic post-operative pain patients and recurrence).

### Monitoring

A number of medical doctors and researchers contributing in the trial will be inventoried in the starting phase of this trial. Participating centres will be visited for explanation trial. Local monitoring is in the hands of the local investigator. The total overview of informed consent forms will be up-to-date at the coordinating research hospital. Monitoring visits will be made by the surgeon-investigator depending on the pace of inclusion.

### Unexpected turns

Serious side effects and incidents, so-called SUSARs and SAEs, should be reported to the central investigator centre, which will make sure these are reported to the ethics committees.

### Interim analysis

No interim analysis is planned.

### Ethical testing

After approval by local institutional review board (Eindhoven), the protocol along with the name of the surgeon from another hospital will be submitted for local recommendations. Any amendments to the protocol will initially be input at IRB Eindhoven by the principal investigator.

### Post-trial care

Care in the protocol exceeds the care customarily spent in time. Since only general accepted techniques are used, the hospital surgeon will be responsible for possible long-term care.

### Co-investigators

Participating co-investigators will be asked to sign an agreement in which the confidentiality of the data as well as their commitment to the trial.

### Publication

The plan is to create three publications. The primary endpoint with short-term results will be processed in one report. This will be one month post-operatively. The the long-term results will be processed in two another report. One report with one-year results and one with two-year result. In the two-year results report, recurrence will be added as secondary outcome measure. Co-investigators with a contribution of at least 10% of the number of patients required will be asked for co-authorship.

## Discussion

This trial is one of the first in its kind, to compare different sorts of mesh types in a randomized controlled setting. Lots of mesh devices are available for herniorraphy made by various companies. Often the choice of the mesh depends on the personal favour of the operating surgeon. The reason to start with this trial is to have a more scientific approach for the decision-making regarding the determination of mesh device.

In this study protocol only the patient is blinded for the treatment. By all means, the operating surgeon cannot be blinded for the type of mesh. The doctor who sees the patients in the outpatient clinic, and fills in forms D2 an D3 can be blind for the treatment type. Because of logistical reasons this is not added to this study protocol. Besides the logistical reasons we think that there is little value in blinding the treating doctor in the outpatient clinic. Observer-bias will be very little because of the straightforward multiple-choice questions in forms D2 and D3.

The results will help to evaluate mesh repair for epigastric an umbilical hernia, and find a surgical device/method that minimizes the complication rate. Besides complication rate, recurrence and costs are taken in account.

## Competing interests

The MORPHEUS trial started as a non-funded trial, because of slow inclusion rate in participating centres due to high device costs; an investigator initiated grand proposal was deposited.

An Investigator Sponsored-Studies (ISS) proposal which is investigator initiated applied for and granted by the Johnson & Johnson Company. This funding was used to create levelling in costs for participating hospitals between the two mesh devices used.

## Authors’ contributions

The idea of starting a trial like this came from SN and JP." to "The concept of the study derived from SN and JP. All authors contributed equally in the creation of this study protocol and will agree to publication. SN and JP are mainly responsible for the revisions made. All authors read and approved the final manuscript.

## Pre-publication history

The pre-publication history for this paper can be accessed here:

http://www.biomedcentral.com/1471-2482/14/33/prepub
